# The Radical Cure of Varicocele, Etc.

**Published:** 1883-11-20

**Authors:** H. C. Boenning

**Affiliations:** Philadelphia


### THE RADICAL CURE OF VARICOCELE BY EXCISION OF THE VENOUS PLEXUS, ILLUSTRATED BY THREE CASES.

BY H. C. BOENNING, M.D., OF PHILADELPHIA.

For the radical cure of varicocele, I have recently performed an operation which, in three cases, has resulted in a speedy, complete cure, and which, if properly done, is superior to the ordinary operation—because, first, it is *simple*, *easily performed*, and the parts under the sense of *sight* as well as feeling; second, it is invariably successful; third, the free drainage prevents absorption of pus or other discharges. It is well known that, as the result of the so-called subcutaneous ligation (intra-scrotal) of the veins, gangrene of the testicle, abscess, sloughs, and pyæmia have occurred, with, in some cases, a fatal result. Free drainage is an acknowledged important factor for the prevention of blood-poisoning, and is as necessary in the operation for the cure of varicocele as in any other wounds. In the ordinary varicocele operations, also, the sense of feeling is entirely and alone relied upon; it is undeniable that, frequently, puncture of the veins, or incomplete ligation of the veins, occurs, resulting in complications interfering with the obliteration of the varicocele; further, in some cases the varicocele, through collateral circulation, returns a few weeks after the so-called radical operation.

The operation of excision is performed as follows:

The parts are shaved; the patient is anæsthetised; an incision is then made through the anterior portion of the scrotum, about three-fourths of an inch to the left of the raphe and about two inches in length, from near the lower portion of the scrotum up; the tissues are divided, layer after layer, upon a director, until the cord, veins and testicle are exposed; the vas deferens is carefully drawn over to the right; the veins are isolated, separated, and an aneurism-needle is passed, armed with a strong catgut or waxed-silked double ligature; the veins are then ligated above and below, the ligatures being placed half an inch or more apart; the veins are then divided midway between the ligatures, as is the thyroid gland in tracheotomy, and the stumps of veins beyond the ligatures are retrenched, if necessary, by the scissors. All the veins, however, should not be ligated; *one or two should be left to return the blood* supplied by the arteries of the cord. The general inference that the veins of the cord are sufficient to carry off the venous blood is incorrect; and, hence, to assist the circulation the above plan should be adopted, lest gangrene ensue, or a chronic congestion of the testicle. After the veins have been ligated, divided and retrenched, the parts should be carefully cleansed, as is the peritoneum after abdominal section, and returned to their places; the ligatures, cut about four inches long, drawn out of the sac at the lower portion, and sutures applied from above downward; three silver wires should be deeply passed *through* the lips of the wound, so as to catch the visceral layer of the tunica vaginalis, leaving a lower aperture of half an inch in length, for drainage.

The parts should be supported either by the scrotal support (see College and Clinical Record, vol. ii, page 264) or towels or oakum. The dressings should be light—carbolized oil, salicylated cotton, lead water and laudanum; or a poultice when indicated; the latter will do good service from about the seventh to the twelfth day. Internally, good nourishment, quinine, or, if fever arises, an aconite fever mixture, is indicated.

From about the third day very mild, warm injections may be used to advantage; thus, one part of carbolic acid to a hundred of water, ten grains of permanganate of potassium to a quart of water, etc., at about ninety-five degrees of heat, should be daily employed in washing out the sac. If the drainage opening show a tendency to close, a small piece of oiled lint may be passed between the lips of the wound. After the tenth day gentle traction may be made upon the ligatures, which all come away before the fourteenth day.

The patient should remain in bed a few days after the ligatures come away. The remaining wound gradually granulates, being eventually a sinus, which, with proper stimulants (one of the best being nitric acid, ten or fifteen drops to the ounce, or a probe coated with the nitrate of silver and passed into the wound daily, every other day, or as circumstances require), heals in a short time. The quantity of fibrin, etc., effused will leave a "lump" in the scrotum, which, however, finally disappears.

The cases I operated upon by this method, as stated before, have made excellent recoveries. In the first case the scrotum was long, pendulous, and instead of making an incision parallel with the raphe, I cut at right angles to it, making two curved incisions, thus removing about two inches of tissue from the front of the scrotum and materially shortening the sac when the sutures were applied. In this case, after the third day all fever subsided, and the ligatures came away, one on the seventh, the other on the thirteenth day. The second case was also successful, but devoid of any especial points of interest.

The third case did not do so well at first. The scrotum was very short, the varicocele slight, but sufficient to keep the patient out of the United States army. At his request I operated. High fever for a week and a slight slough of the tunica vaginalis occurred; then the case assumed a better character, and is now nearly well, nothing but a small opening still remaining.

After the patients leave their bed, I urge them to wear a good suspensory for a few months, after which they cast it aside. All of my cases have been up, attending to their various pursuits, by the sixteenth day; in fact, the last two were enabled to resume their work before the fourteenth day.

The advantages of the operation, briefly, are these: simplicity, certainty of success and rapidity of cure—to say nothing of the free drainage and the free exposure of the parts involved; hence, the impossibility for any surgeon who knows anatomy to do other than what the case demands.—*Philadelphia Medical Times.*

